# A novel CD123-targeted therapeutic peptide loaded by micellar delivery system combats refractory acute myeloid leukemia

**DOI:** 10.1186/s13045-021-01206-y

**Published:** 2021-11-13

**Authors:** Shilin Xu, Meichen Zhang, Xiaocui Fang, Jie Meng, Haiyan Xing, Doudou Yan, Jian Liu, Yanlian Yang, Tao Wen, Weiqi Zhang, Jianxiang Wang, Chen Wang, Haiyan Xu

**Affiliations:** 1 Institute of Basic Medical Sciences, Chinese Academy of Medical Sciences & Peking Union Medical College, Beijing, 100005 China; 2 CAS Key Laboratory of Biological Effects of Nanomaterials and Nanosafety, CAS Key Laboratory of Standardization and Measurement for Nanotechnology, CAS Center for Excellence in Nanoscience, National Center of Nanoscience and Technology, Beijing, 100190 China; 3 University of the Chinese Academy of Sciences, Beijing, 100190 China; 4 State Key Laboratory of Experimental Hematology, Institute of Hematology and Blood Diseases Hospital, Chinese Academy of Medical Sciences & Peking Union Medical College, Tianjin, 300020 China

**Keywords:** Acute myeloid leukemia, CD123, Antagonistic peptide, Micelle, Targeting

## Abstract

Acute myeloid leukemia (AML) is a common malignant heterogeneous hematopoietic disease with very low average 5-year survival rate due to the refractory feature and high rate of relapse. CD123 is highly expressed on multiple types of AML cells, especially leukemia stem cells, and closely associated with the poor prognosis of AML. Aiming to meet the urgent demand to targeted therapeutics for the refractory AML patients, herein we synthesize a CD123 antagonistic peptide (PO-6) loaded in nanomicelles (^m^PO-6), and investigated its therapeutic effect and pharmacokinetics on a lab-established refractory AML mice model (AE & CKIT^D816V^). It is shown that the PO-6 can effectively bind to the CD123^+^ AML cells and the micellar formulation ^m^PO-6 increases the dissolution stability and the specific binding capacity. When injected intravenously, ^m^PO-6 significantly prolongs the survival of the refractory AML mice by interfering CD123/IL-3 axis, evidenced by the down regulation of phosphorylation of STAT5 and PI3K/AKT and the inhibition of activated NF-κB in the nucleus, as well as by the analysis results of next generation RNA-sequencing (RNA-seq) with the bone marrow of the AML mice. The antagonistic effect leads to the significantly reduction of AML cells infiltration in the bone marrow of the AML mice. In conclusion, ^m^PO-6 could provide a potent antagonistic therapeutic approach for targeted treatment of AML.

**Supplementary Information:**

The online version contains supplementary material available at 10.1186/s13045-021-01206-y.

## To the Editor,

Acute myeloid leukemia (AML) is a common malignant heterogeneous hematopoietic disease with very low average 5-year survival of 25% due to the refractory feature and high rate of relapse [1, 2]. CD123 is a membrane protein expressed in ~ 80% of AMLs as well as leukemia stem cells and is closely related with the prognosis of AML patients [3, 4]. Although several anti-CD123 antibody-based medicines have shown significant therapeutic effects in animal models [5–7], yet encouraging results have not been achieved rigorously from the clinical trials [8, 9].

**Fig. 1:**
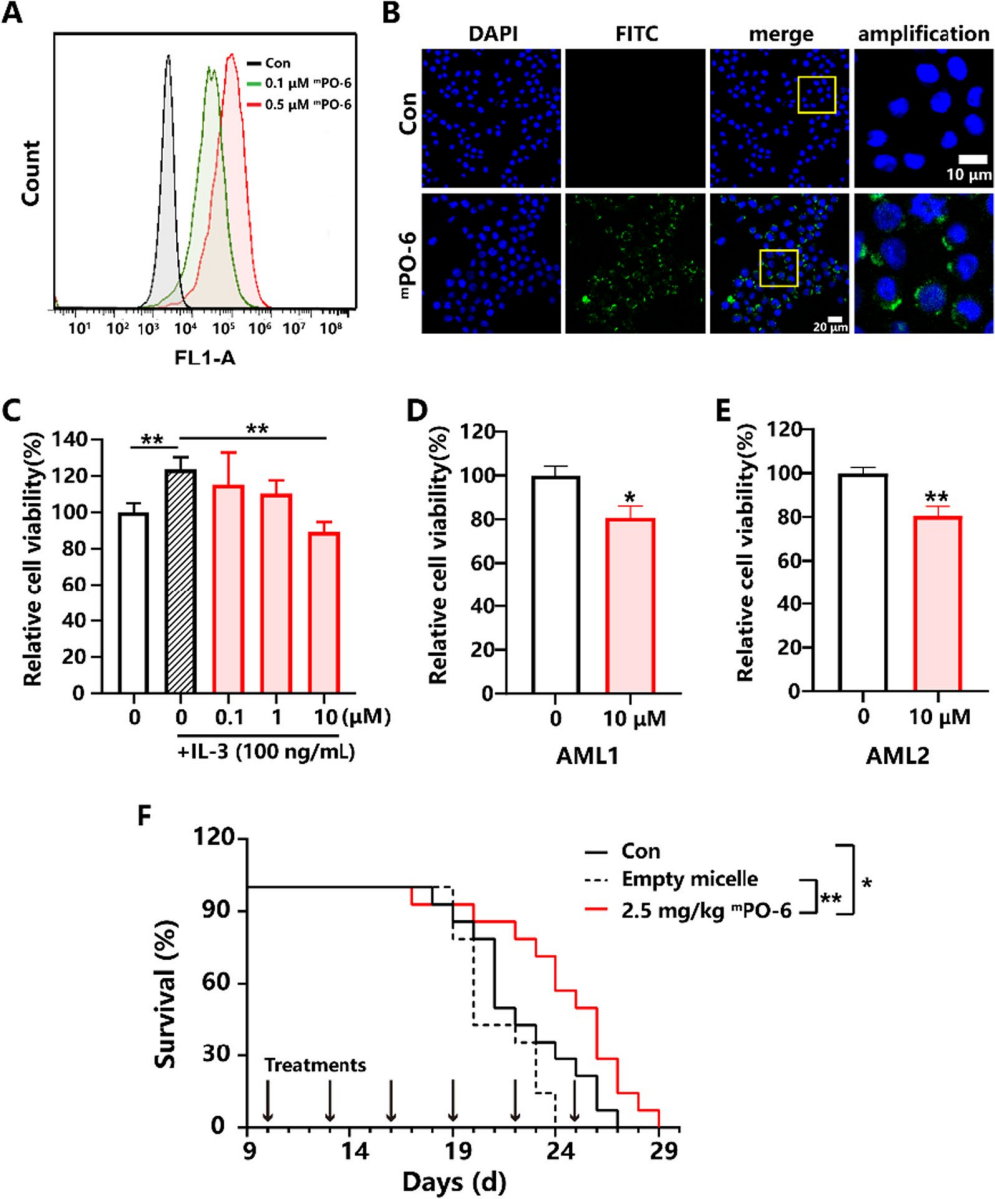
**A** Affinity of ^m^PO-6 to MOLM-13 cells incubated at 0.1 and 0.5 μM for 0.5 h. **B** Images of ^m^PO-6 bound with MOLM-13 cells at 0.1 μM for 0.5 h obtained from the laser confocal microscopy. **C** Effects of ^m^PO-6 on the cell viability in the presence of IL-3 (*n* = 4). **D** and **E** Effects of ^m^PO-6 on the cell viability of the primary AML blasts. **F** The treatment of ^m^PO-6 prolonged the refractory AML mice median survival significantly (*n* = 14). *P < 0.05, **P < 0.01

Antagonistic peptides provide a promising venue to develop protein-targeting therapeutics for AML treatments. In this work, a novel CD123 antagonistic peptide (PO-6) has been obtained based on screening of the cell culture from a group of de novo designed peptides targeting to various segments of the CD123 protein [10] (Table S1). The PO-6 was chemically synthesized and could effectively bind to the CD123^+^ AML cell line MOLM-13 cells in a concentration-dependent manner while weakly bind to the CD123^−^ AML cell line HL-60 and chronic myeloid leukemia cell line K562 cells (Additional file 1: Fig. S1), showing its recognition specificity to CD123. The PO-6 was further assembled with amphiphilic polymeric molecules to form peptide-loading micelles (^m^PO-6) with the average diameter of 38 nm (Additional file 1: Fig. S2). The ^m^PO-6 could bind to the MOLM-13 cells as well (Fig. 1A) and achieve a higher binding amount to the MOLM-13 cells and distribute more homogenously on the cell membrane (Fig. 1B) than PO-6 (Additional file 1: Fig. S3), because the polymeric micelles improved the dissolution stability of PO-6 in physiological conditions. To verify its antagonistic effect, ^m^PO-6 was incubated with MOLM-13 cells in the presence of IL-3 that is the ligand of CD123 as well as with the primary blasts from two patients diagnosed as refractory AML. Results showed that ^m^PO-6 could competitively bind to the extracellular N-terminal domain of CD123 on the MOLM-13 cells, which is the IL-3 binding site (Additional file 1: Fig. S4). The IL-3 mediated activation of MOLM-13 cells was effectively inhibited by ^m^PO-6 (Fig. 1C), which evidenced that ^m^PO-6 had the expected antagonistic function of interrupting the axis of CD123/IL-3. Moreover, ^m^PO-6 could bind to the primary blasts expressing CD123 (Additional file 1: Fig. S5) and inhibit the viability of the cells (Fig. 1D, E). For in vivo study, a refractory AML mice model was established by intravenously injecting AE & CKIT^D816V^ cells expressing CD123 (Additional file 1: Fig. S6). On the model that did not respond to cytarabine hydrochloride or homoharringtonine (Additional file 1: Fig. S7), ^m^PO-6 of 2.5 mg/kg significantly prolonged the median survival (Fig. 1F) in reference to the control or empty micelle group, displaying a very encouraging therapeutic effect.

**Fig. 2:**
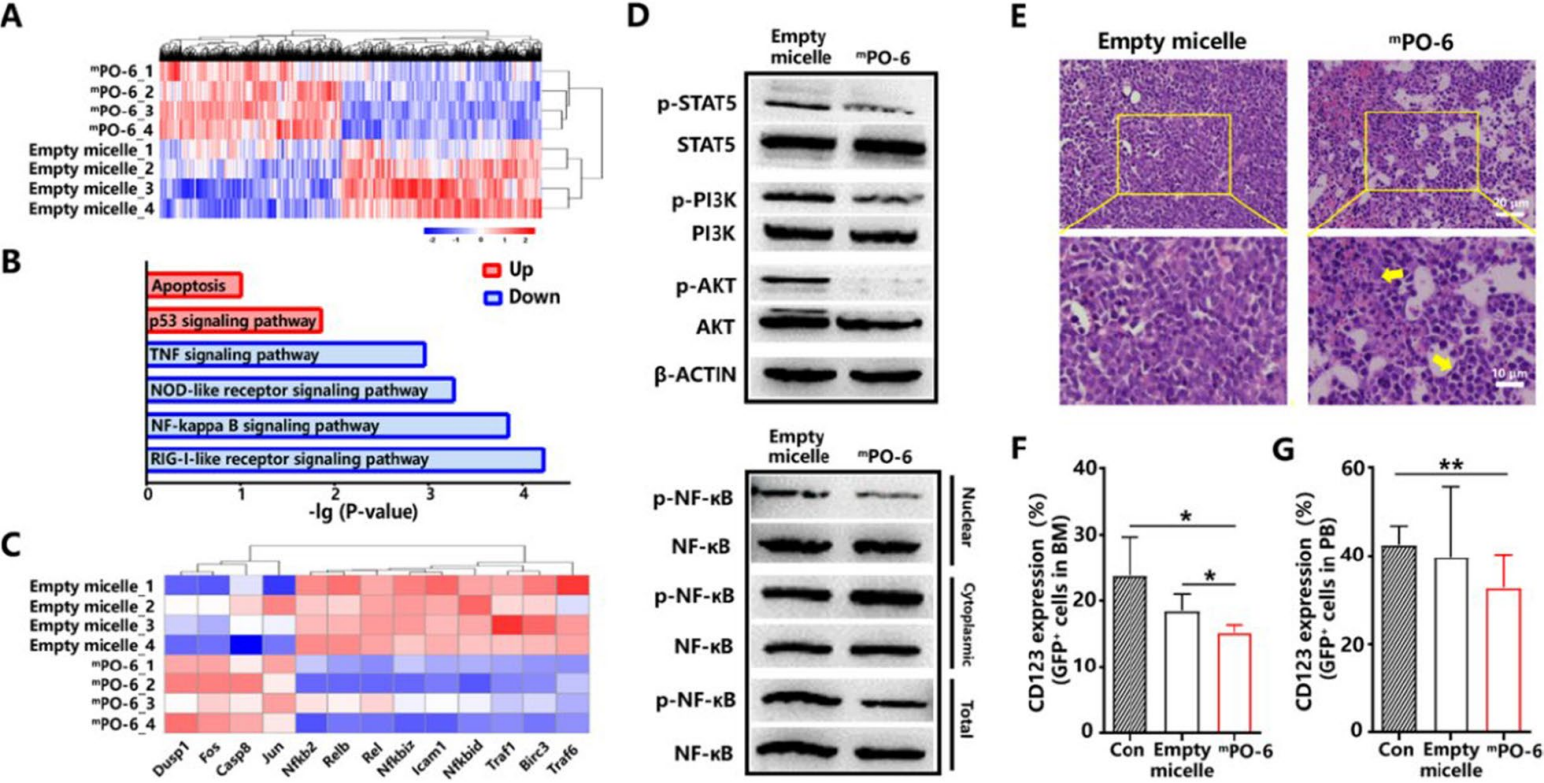
**A** Hierarchical clustering of genes expression of the AML cells in the BM for the group of empty micelle and ^m^PO-6 at 24 h post injection. Blue and red colors represented down-regulated or up-regulated genes respectively (*n* = 4). **B** Cluster of genes that were analyzed by Kyoto Encyclopedia of Genes and Genomes (KEGG) for identification of the affected biological processes with the treatment of ^m^PO-6. **C** Genes were alternated significantly in the BM related to the CD123/IL-3 axis (*n* = 4). **D** The phosphorylation of STAT5, PI3K/AKT and the expression of NF-κB in the nucleus, cytoplasm and total protein in the group of empty micelle and ^m^PO-6. Leukemia cells were separated from the BM of the AML mice scarified 24 h after the third i.v. injection of empty micelles or ^m^PO-6. **E** Histologic sections of BM in the mice stained with H&E, the yellow arrows pointed normal cells. The expression of CD123 on AML cells in the BM **F** and PB **G** was reduced by ^m^PO-6 (*n* = 4). *P < 0.05, **P < 0.01

Next generation RNA-sequencing was performed with AML cells separated from the bone marrow (BM) of the AML mice post three intravenous administrations of ^m^PO-6 or empty micelles. Results showed that ^m^PO-6 induced 1716 genes down-regulated and 1556 ones up-regulated (Fig. 2A), and the differentially expressed genes were involved in the IL-3-mediated signaling pathways (Fig. 2B). Furthermore, the genes related to NF-κB, TNF, RIG-I-like and NOD-like receptor were enriched in the negative regulation while those linked to the signaling pathways of p53 and apoptosis were enriched in the positive regulation (Fig. 2C). Western blotting analysis showed that ^m^PO-6 could significantly inhibit the phosphorylation of STAT5, PI3K/AKT, and NF-κB in the nucleus in the BM (Fig. 2D), which are the downstream signaling proteins of CD123/IL-3 [11, 12]. Moreover, there were less infiltrating AML cells (Fig. 2E) observed and the lower level of CD123 (Fig. 2F) detected in the BM and peripheral blood (Fig. 2G) of the AML mice received the ^m^PO-6 treatment compared with those received empty micelles, clearly displaying the therapeutic effects of ^m^PO-6 at both molecular and histological level. When injected to healthy mice, ^m^PO-6 rapidly distributed to the liver, lung and kidney (Additional file 1: Fig. S8), and was mainly excreted through kidney (Additional file 1: Fig. S8). Additionally, ^m^PO-6 of 10 mg/kg did not induce significant changes in the number of white blood cells, red blood cells and platelets of the mice at 24 h post injection (Additional file 1: Fig. S9; Additional file 2).

In summary, we report a novel and chemically synthesized peptide with antagonistic function towards CD123. The peptide in its micellar formulation displayed significant anti-leukemia activities in the refractory AML mice, providing an effective and safe therapeutics to the refractory AML treatments.

## Supplementary Information

**Additional file 1**. **Supplementary material and methods**. ** Fig. S1.** Affinity of PO-6 and ^m^PO-6 to different leukemia cells. **Fig.S2.** The TEM image of ^m^PO-6. **Fig. S3.** Confocal microscopy observation of PO-6 binding with MOLM-13 cells.** Fig. S4.** ^m^PO-6 inhibited the antibodies binding to CD123. **Fig. S5.** Affinity of ^m^PO-6 to the AML blasts.** Fig. S6. **CD123 expression on GFP^+^ cells in BM of AE & CKIT^D816V^ mice.** Fig. S7. **Therapeutic efficacy of Ara-C or HHT in AE & CKIT^D816V^ mice. **Fig. S8.** The fluorescent representative imaging of main organs at designated time points after ^m^PO-6-FITC injection in healthy mice.** Fig. S9.** Acute toxicity evaluation of ^m^PO-6 in healthy mice. **Table S1.** Human and mouse CD123 alignment of the extracellular amino acid sequences.

**Additional file 2.** Materials and Methods.

## Data Availability

The datasets used and/or analyzed during the current study are available from the corresponding author on reasonable request.

## Abbreviations

AML: Acute myeloid leukemia
BM: Bone marrow
PB: Peripheral blood
KEGG: Kyoto encyclopedia of genes and genomes
