# Supplementary material for: A novel CD123-targeted therapeutic peptide loaded by micellar delivery system combats refractory acute myeloid leukemia
Source: J Hematol Oncol. 2021 Nov 13;14:193. doi: 10.1186/s13045-021-01206-y (PMC8590286; doi:10.1186/s13045-021-01206-y)
Supplement: Supplementary file 1 — Additional file 1. Supplementary material and methods. Fig. S1. Affinity of PO-6 and mPO-6 to different leukemia cells. Fig.S2. The TEM image of mPO-6. Fig. S3. Confocal microscopy observation of PO-6 binding with MOLM-13 cells. Fig. S4. mPO-6 inhibited the antibodies binding to CD123. Fig. S5. Affinity of mPO-6 to the AML blasts. Fig. S6. CD123 expression on GFP+ cells in BM of AE & CKITD816V mice. Fig. S7. Therapeutic efficacy of Ara-C or HHT in AE & CKITD816V mice. Fig. S8. The fluorescent representative imaging of main organs at designated time points after mPO-6-FITC injection in healthy mice. Fig. S9. Acute toxicity evaluation of mPO-6 in healthy mice. Table S1. Human and mouse CD123 alignment of the extracellular amino acid sequences. [file 13045_2021_1206_MOESM1_ESM.docx]

**Additional file 1.** Supplementary material for

**A novel CD123-targeted therapeutic peptide loaded by micellar delivery system combats refractory acute myeloid leukemia**

Shilin Xu^1^, Meichen Zhang^1^, Xiaocui Fang^2, 3^, Jie Meng^1^, Haiyan Xing^4^, Doudou Yan^1^, Jian Liu^1^, Yanlian Yang^2, 3^, Tao Wen^1^, Weiqi Zhang^1^, Jianxiang Wang^4*^, Chen Wang^2, 3*^, Haiyan Xu^1*^

*Corresponding author. Email: [xuhy@pumc.edu.cn](mailto:xuhy@pumc.edu.cn), [wangch@nanoctr.cn](mailto:wangch@nanoctr.cn), [wangjx@ihcams.ac.cn](mailto:wangjx@ihcams.ac.cn)

Supplementary Material

**Materials and Methods**

**Flow cytometry assay**

***The binding of ^m^PO-6 to AML blasts:***

The AML blasts of 2×10^5^ were incubated with FITC-conjugated anti-human CD123 antibody (Biolegend, San Diego, CA) at room temperature or FITC-labeled ^m^PO-6 (0.5 μM) at 37 ℃ for 0.5 h. Resulting cells were subjected to C6 Accuri® flow cytometer and 1×10^4^ cells were collected. Acquired data were analyzed by CFlow Plus software.

***Competitive binding of ^m^PO-6 with CD123 Antibody:***

MOLM-13 cells of 4×10^5^/mL were pretreated with different concentrations (0-20 μM) of ^m^PO-6 for 2 h at 37 °C, followed by staining with PE-CF594-conjugated anti-CD123 7G3 and Alexa Fluor 647-conjugated anti-CD123 9F5 (BD Bioscience, USA) for 0.5 h at the room temperature. Then the binding of ^m^PO-6 and antibodies 7G3 and 9F5 on the surface of the cells were analyzed by C6 Accuri® flow cytometer.

**Survival experiments of cytarabine hydrochloride or homoharringtonine in the AML mice**

*The regimen of cytarabine hydrochloride (Ara-C):* On day 9 after AE & CKIT^D816V^ cells implantation, the mice were randomly divided into 4 groups (n=9) including Con (5% glucose), 25, 50 and 100 mg/kg Ara-C. All mice were treated with daily intraperitoneal (i.p.) injections for 4 days and stopped. The survival of the mice was recorded every day.

*The regimen of* *homoharringtonine (HHT):* On day 9 after AE & CKIT^D816V^ cells implantation, the mice were randomly divided into 2 groups (n=8) and i.p. injected with 5% glucose and 250 μg/kg HHT. The administration was given every 2 days. The survival of the mice was recorded every day. The survival curves of Ara-C or HHT on AE & CKIT^D816V^ mice model were showed in Figure S7.

**Figures**


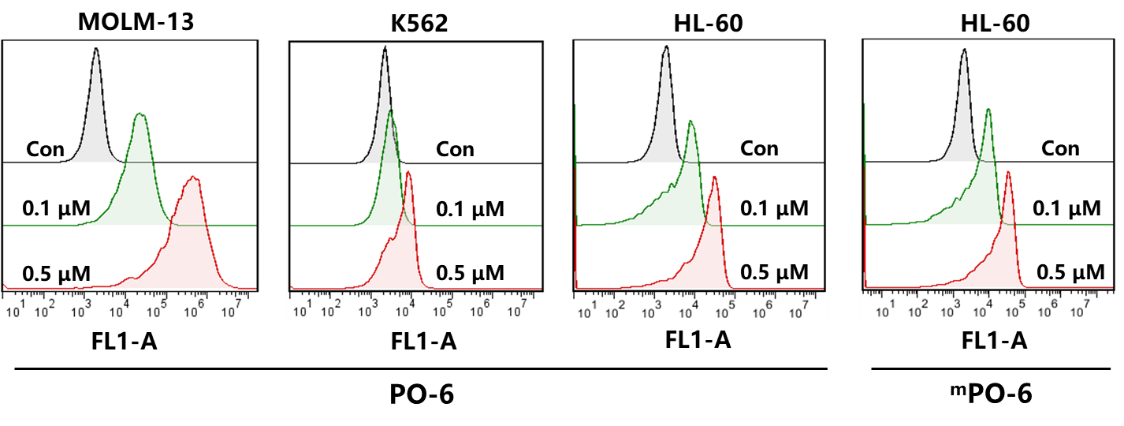


Fig. S1. Affinity of PO-6 and ^m^PO-6 to different leukemia cells. The MOLM-13, K562 or HL-60 cells was incubated with PO-6 or ^m^PO-6 at 0.1 and 0.5 μM for 0.5 h. Resulting cells were collected and subjected to C6 Accuri® flow cytometer.


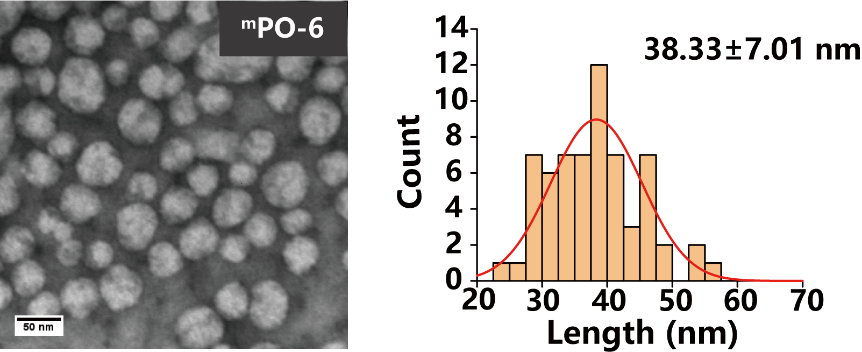


Fig. S2. The TEM image of ^m^PO-6. Morphology of ^m^PO-6 observed by TEM and the size distribution of ^m^PO-6 was acquired by measuring at least 60 particles.


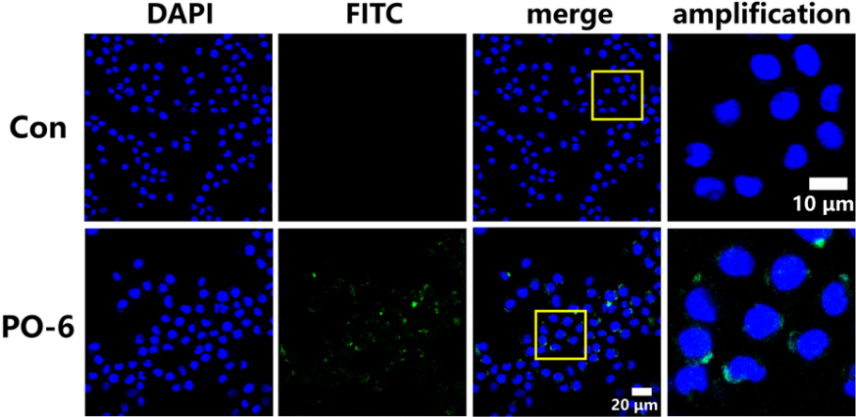


Fig. S3. Confocal microscopy observation of PO-6 binding with MOLM-13 cells. The binding of PO-6 with MOLM-13 cells at 0.1 μM for 0.5 h observed by laser confocal microscopy.


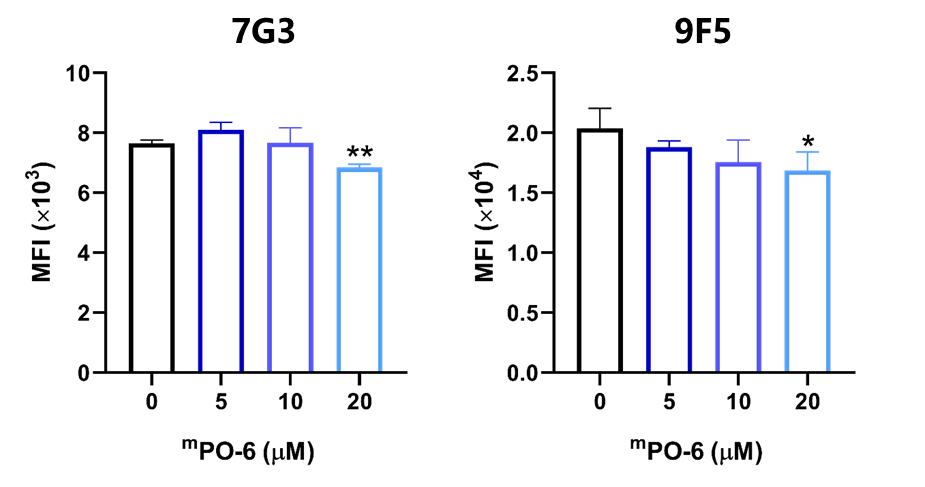


Fig. S4. ^m^PO-6 inhibited the antibodies binding to CD123. The mean fluorescence intensity (MFI) of different clones of CD123 antibody (7G3 and 9F5) on MOLM-13 cells after pretreated with ^m^PO-6 at different concentrations (0-20 μM) for 2 h (n=3). *P<0.05, **P<0.01.


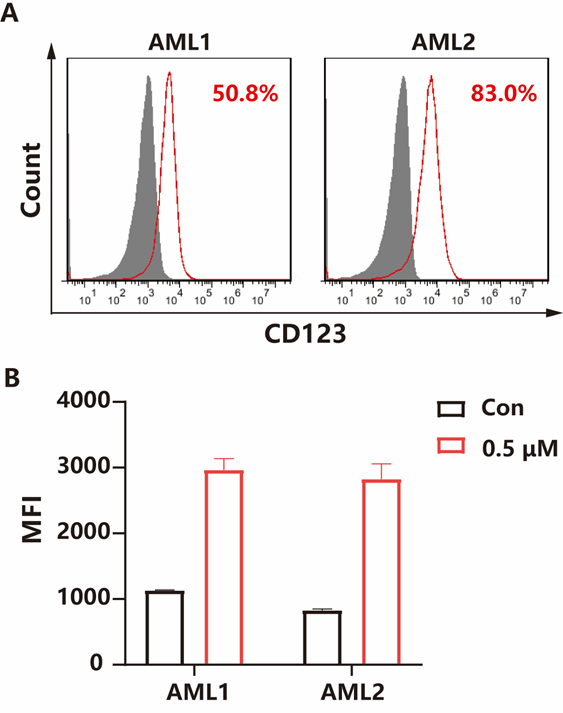


Fig. S5. Affinity of ^m^PO-6 to the AML blasts. (A) The CD123 level on the AML blasts. (B) The binding amount of ^m^PO-6 with AML blasts at 0.5 μM for 0.5 h.


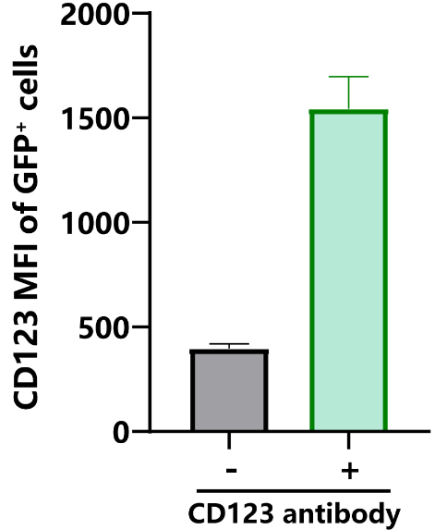


Fig. S6. CD123 expression on GFP^+^ cells in BM of AE & CKIT^D816V^ mice (n=4). The cells isolated from the BM of AML mice were incubated with PE-labeled anti-mouse CD123 antibody in PBS for 0.5 h at room temperature and detected the level of CD123 by C6 Accuri^®^ flow cytometer.


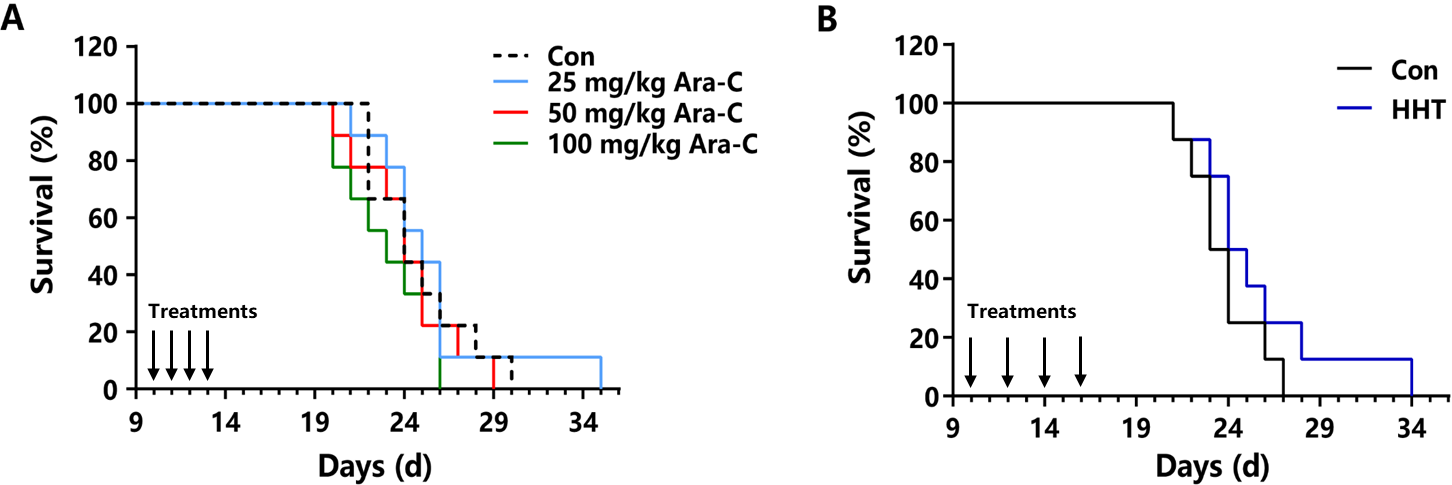


Fig. S7. Therapeutic efficacy of Ara-C or HHT in AE & CKIT^D816V^ mice. (A) The median survival of AML mice treated with Ara-C was not prolonged compared with the Con group (n=9). (B) HHT administration did not prolong the AML mice median survival significantly (n=8). On day 9 after AE & CKIT^D816V^ cells implantation, AML mice were treated with i.p. injection of 5% glucose, Ara-C (25, 50 and 100 mg/kg) or HHT (250 μg/kg) as the regimens described.


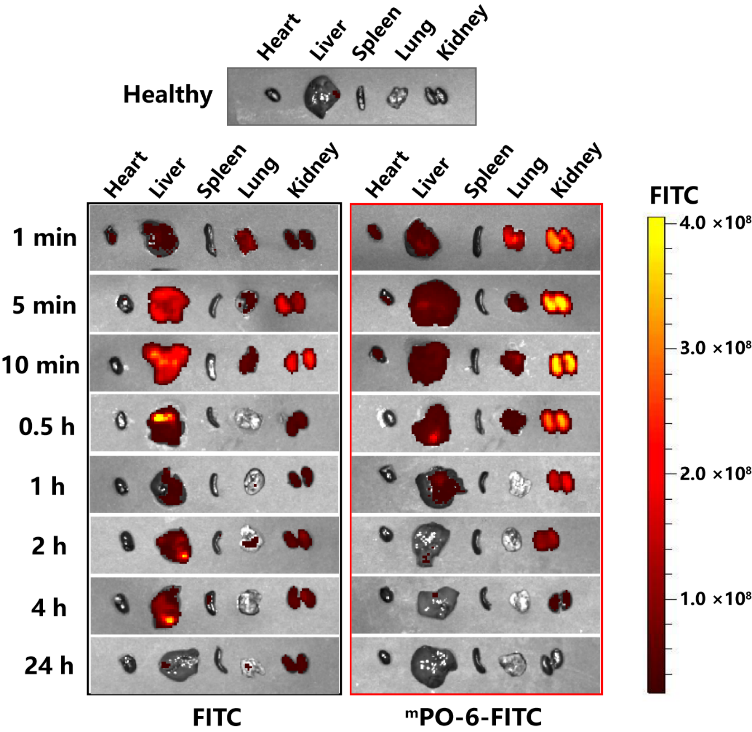


Fig. S8. The fluorescent representative imaging of main organs at designated time points after ^m^PO-6-FITC injection in healthy mice. FITC-labeled ^m^PO-6 (Soluplus: 50 mg/kg, PO-6: 10 mg/kg) was intravenously injected into healthy mice. The free FITC of the same concentration to ^m^PO-6-FITC was set as control. The mice were sacrificed at designated time points, and the heart, liver, spleen, lung and kidney were collected for ﬂuorescence imaging using Xenogen IVIS spectrum (Caliper Life Science, USA). The ﬂuorescence was measured at an excitation wavelength of 488 nm and an emission wavelength of 525 nm.


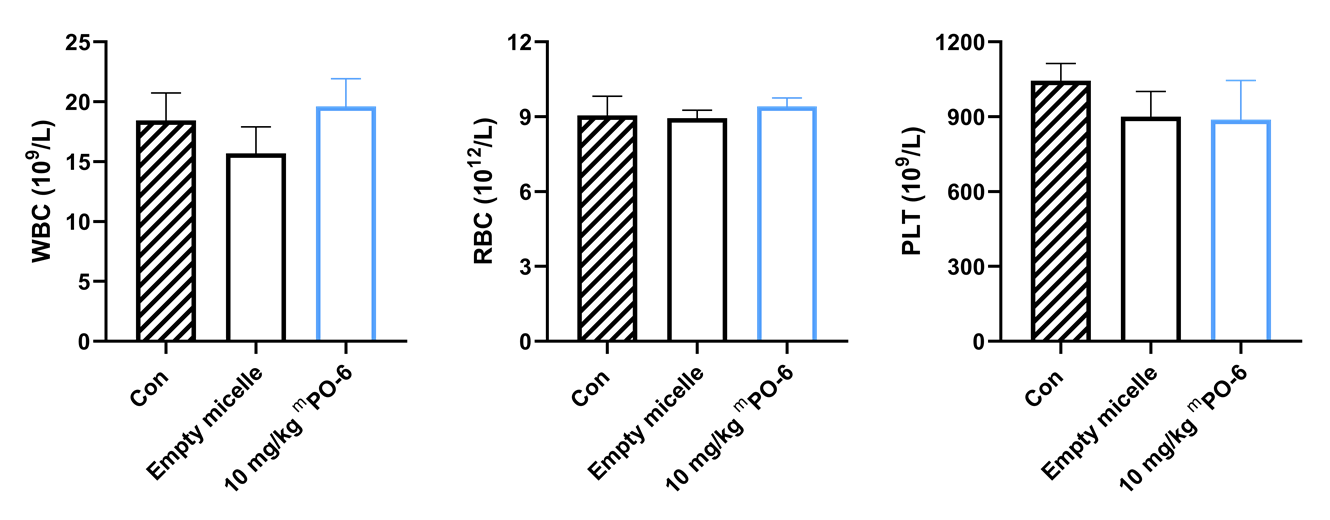


Fig. S9. Acute toxicity evaluation of ^m^PO-6 in healthy mice. Healthy C57 mice were randomly divided into 3 groups, including control, empty micelle and 10 mg/kg ^m^PO-6. The number of white blood cells (WBC), red blood cells (RBC) and platelets (PLT) in the peripheral blood of healthy mice were determined by the auto hematology analyzer BC-5120 (Mindray Bio-Medical Electronics Co., Ltd., China) at 24 h post injection (n=5).

**Tables**

**Table S1. Human and mouse CD123 alignment of the extracellular amino acid sequences**

|  | Identities: 91 (30%)  (marked in red color) | Chemically similar: 41 (14%)  (marked in green color) |
| --- | --- | --- |
| Human 26-69  Mouse 33-80 | PITNLRMKAKAQQLTWDLNRNV----TDIE-CVKDAD-----YSMPAVNNSYCQ  PIQNLHI-DPAHYT-LSWDPAPGADITTGAFCRKGRDIFVWADPGL----ARCS | |
| Human 70-119  Mouse 81-131 | FGAISLCEVTNYTVRVA-NPPFSTWILFP---ENS-GKPWAGAENLTCW-I-HDVDF  FQSLSLCHVTNFTVFLGKDRAVAGSIQFPPDDDGDHEAAAQDLRCWVHEGQ- | |
| Human 120-156  Mouse 132-178 | LSCSWAV-GPGAPADVQY------------DLY-LNVANRRQQYECLHYKT-------------  LSCQWERGPKATGDVHYRMFWRDVRLGPAHNR------ECPHYHSLDVNTAGP | |
| Human 157-201  Mouse 179-229 | DAQGTRI-GCRFDDISRLSSGSQSSH-------------ILVRGRSA-AFG-IPCT-DKFVV  APHGGHEGCTLDLDTVLGSTPNSPDLVPQVTITVNGSGRAGPVPCMDNTVD | |
| Human 202-251  Mouse 230-280 | FSQ-IE-ILTPPNMTAKCNKTHSFMHWKMRSHFNRK—FRYELQIQKRMQPVIT | |
|  | LQRAEVLAPPT-LTVECNGSEAHARWVA-RNRFHHGLLGYTLQVNQSS-RSEPQ | |
| Human 252-296  Mouse 281-326 | EQVRDRTSFQLL-NPGTYTVQIRARERVYEF--LSAWSTPQRFECDQE | |
|  | EYNVSIPH-FWVPNAGAISF-RVKSRSEVYPRKLSSWSEAWGLVCPPE | |

**Source of sequence alignment:** https://www.ncbi.nlm.nih.gov
