# Supplementary material for: A novel CD123-targeted therapeutic peptide loaded by micellar delivery system combats refractory acute myeloid leukemia
Source: J Hematol Oncol. 2021 Nov 13;14:193. doi: 10.1186/s13045-021-01206-y (PMC8590286; doi:10.1186/s13045-021-01206-y)
Supplement: Supplementary file 2 — Additional file 2. Materials and Methods. [file 13045_2021_1206_MOESM2_ESM.docx]

**Additional file 2.** **Materials and Methods**

**Peptide PO-6**

We screened the peptide through a cell-based selection process by screening of fragments of targeted proteins to obtain peptide ligands with high binding affinities. This fragmental screening process was supported by the principles of inter-amino-acid recognition revealed by binding energies between homogeneous oligopeptides in our previous study for unraveling both main-chain-related and side-chain-related interactions originating from the chemical structures of the 20 common naturally occurring amino acids [1], and has been illustrated in our previous studies [2-4]. A peptide PO-6 (DDDYDTRAQGTDIGCDFRRISDDD) was identified by the cell-based selection process and was synthesized and purchased from Guoping Pharmaceutical Co., Ltd (Anhui, China).

**Ethics approval**

The bone marrow samples were obtained from two patients diagnosed as refractory AML, who were enrolled in the Institute of Hematology and Blood Diseases Hospital, Chinese Academy of Medical Sciences. This subject was approved by the ethical committee in the Institute of Hematology and Blood Diseases Hospital and all procedures in accordance with the Declaration of Helsinki.

**Cell culture**

The human acute myeloid leukemia cell line MOLM-13 was offered by Prof. Min Wang, Institute of Hematology and Blood Diseases Hospital (Tianjin, China). The human chronic myeloid leukemia cell line K562 and human acute myeloid leukemia cell line HL-60 was purchased from the Cell Resource Center of Chinese Academy of Medical Sciences (Beijing, China). All cells were cultured in RPMI 1640 medium (Hyclone Thermo Scientific, USA) supplemented with 10% fetal bovine serum (FBS, Gibco, Life Technologies, USA) and phenol red, 100 U/mL penicillin (Hyclone), 100 U/mL streptomycin (Hyclone). Cells were cultured at 37°C in a humidified atmosphere containing 5% CO_2_.

**Flow cytometry assay**

MOLM-13, HL-60 and K562 cells of 4×10^5^/mL were incubated with FITC-labeled PO-6 or ^m^PO-6 at 0.1 μM and 0.5 μM for 0.5 h. After twice washed with PBS, subjected to C6 Accuri^®^ flow cytometer (Accuri Cytometers, Ann Arbor, MI) and 1×10^4^ cells were collected. Acquired data were analyzed by CFlow Plus software.

**Preparation and characterization of PO-6-loaded micelles**

PO-6-loaded micelles (^m^PO-6) was prepared by a one-step self-assembly method. Briefly, FITC-labeled, unlabeled PO-6, and polyvinyl caprolactam-polyvinyl acetate-polyethylene glycol graft copolymer (Soluplus^®^, BASF SE, Germany) was dissolved in sterile PBS solution separately. Then the solution of Soluplus was mixed with the solution of PO-6 at a mass ratio of 5:1. The concentration of Soluplus in the PBS solution was 0.5 mg/mL that is higher than its critical micelle concentration (CMC) of 7.6×10^-3^ mg/mL [5], thus the polymer molecules formed empty micelles. To acquire the particle size distribution of ^m^PO-6 observed by transmission electron microscope (TEM, Hitachi 1400 plus, Japan), the size of at least 60 particles was measured using the Image J software.

**Laser-scanning confocal microscopy**

MOLM-13 cells of 4×10^5^/mL were incubated with FITC-labeled PO-6 or ^m^PO-6 at 0.1 μM for 0.5 h at 37°C. The cells were then washed with PBS followed by fixation with 4% paraformaldehyde for 0.5 h. The fixed cells were centrifuged at 1000 rpm for 5 min by table-top low speed centrifuge (ROTOFIX 32A, Hettich, Germany). The nucleus was stained with DAPI. Green fluorescence of FITC and blue fluorescence of DAPI were acquired by confocal microscopy (OlympusFV1000, Center Valley, PA). Images were analyzed by Volocity-5 software (Perkin Elmer).

**CCK-8 assay**

MOLM-13 cells of 4×10^5^/mL were seeded onto 96-well culture plates containing 0.5% FBS RPMI 1640 and starved overnight, then replaced with 10% FBS RPMI 1640. The cells were pretreated with ^m^PO-6 (0.1-10 μM) for 4 h, followed by the supplementation of IL-3 (PeproTech, USA) at 100 ng/mL and co-incubated for 20 h at 37℃. The bone marrow samples from AML patients were treated with ^m^PO-6 (10 μM) at 37℃ with 15% FBS IMDM medium (Gibco, USA) for 24 h. After incubation, CCK-8 was used to measure the cells viability. In brief, 10 μL of the Cell Counting Kit-8 (Dojindo Laboratories, Japan) agent in 100 μL RPMI 1640 medium was added to each well, followed by 2 h incubation. The optical density (OD) value was read at 450 nm and 630 nm using a microplate reader (Thermo, USA). The viability percentage of untreated cells as control group was set as 100%, and the relative cell viability was the ratio of that for treatment groups in reference to that for control group.

**AE & CKIT^D816V^ leukemia mouse model establishment**

The AML1-ETO (AE) & CKIT^D816V^ refractory leukemia mouse model was established following the procedure reported previously [6]. In brief, female C57Bl/6 mice (6-8 weeks old) were intravenously injected thawed 1×10^6^ of splenic AE & CKIT^D816V^ cells (GFP^+^) following the X-ray irradiation at a dose of 450 cGy. The mice were sacrificed when they became moribund, and splenic cells were isolated and injected intravenously into secondary recipients for *in vivo* experiments.

**Survival experiment**

On day 9 after AE & CKIT^D816V^ cells implantation, the mice were randomly divided into 3 groups (n=14), including Con (PBS solution), empty micelle (12.5 mg/kg of Soluplus), and ^m^PO-6 (PO-6: 2.5 mg/kg, Soluplus: 12.5 mg/kg). The administration was given every 3 days, three i.v. injections followed by three subcutaneous injections. The mice were weighed every other day and the death of the mice was recorded.

**Therapeutic effect experiments**

On day 7 after AE & CKIT^D816V^ cells implantation, the mice were randomly divided into 3 groups (n=4): Con (PBS solution), empty micelle (12.5 mg/kg of Soluplus) and ^m^PO-6 (PO-6: 2.5 mg/kg, Soluplus: 12.5 mg/kg). All the mice were scarified 24 h after the third i.v. injection. The bone marrow (BM) samples were fixed with 4% paraformaldehyde and embedded in paraffin. Then tissue sections were prepared and stained by hematoxylin and eosin (H&E). Cells isolated from the peripheral blood (PB) and BM of AML mice were incubated with PE-labeled anti-mouse CD123 antibody (Biolegend, San Diego, CA) in PBS for 0.5 h at room temperature and subjected to C6 Accuri^®^ flow cytometer to detect the level of CD123.

**RNA-sequencing analysis**

On day 9 after AE & CKIT^D816V^ cells implantation, C57Bl/6 mice were randomly divided into 2 groups (n=4): empty micelle (12.5 mg/kg of Soluplus) and ^m^PO-6 (PO-6: 2.5 mg/kg, Soluplus: 12.5 mg/kg). The mice were administrated by i.v. injection every 3 days. At 24 h post the third injections, mice were sacrificed. GFP^+^ cells were sorted from the single cell suspension of BM (Moflow-XDP, Beckman, USA) and preserved in Trizol solution (Sigma-Aldrich, USA). The Next generation of RNA-sequencing was performed by Novogene (Beijing, China). Differences in gene expression between the group of empty micelle and ^m^PO-6 were analyzed by DESeq2R package (1.10.1) (P<0.05). The processed data were used for KEGG (Kyoto Encyclopedia of Genes and Genomes) annotation enrichment analysis using cluster Profiler R package.

**Western blotting**

As for the preparation of protein samples from the in vivo experiment, the AML cells sorted from the BM of AE & CKIT^D816V^ mice which were sacrificed at 24 h post the third injections were washed and lysed. The lysates were centrifuged at 12,000 rpm, 4℃ for 15 min. Protein of 20 μg for each group was loaded and separated on 12% polyacrylamide gels, and then transferred to PVDF membranes (0.45 μm; Millipore, Bedford, MA). The extraction of cytoplasmic protein and nuclear protein was used by Nuclear and Cytoplasmic Protein Extraction Kit (Beyotime Biotechnology, China). The membrane was incubated with primary antibodies of STAT5, phosphorylated-STAT5, PI3K, phosphorylated-PI3K, AKT, phosphorylated-AKT, NF-κB and phosphorylated-NF-κB (Cell Signaling Technology, Beverly, MA, USA) at 4℃ overnight. After washed with TBST three times, the membrane was treated with HRP Goat anti-mouse IgG (Biolegend, San Diego, CA) or HRP Goat anti-rabbit IgG (Jackson ImmunoResearch, USA) for 1.5 h at room temperature. The membranes were imaged through gel imaging system (Tanon Science & Technology Co., Ltd., Shanghai, China).

**Animal ethics statement**

All the animal experiments reported herein were carried out in accordance with the approved guideline and approved by the committee on the Animal Care and Use of Institute of Basic Medical Sciences, Chinese Academy of Medical Sciences & Peking Union Medical College.

**Statistical analysis**

Results are reported as the mean ± SD unless otherwise indicated. Statistical analysis of the data was performed using GraphPad Prism 8.0 software. Differences between groups were analyzed by Student’s t-test and one-way ANOVA statistical analysis. Survival curves were compared by the log-rank test. P <0.05 are indicated by “*****” and P <0.01 are indicated by “******”.

**References**

[1] Du H, Hu X, Duan H, Yu L, Qu F, Huang Q, Zheng W, Xie H, Peng J, Tuo R, Yu D, Lin Y, Li W, Zheng Y, Fang X, Zou Y, Wang H, Wang M, Weiss PS, Yang Y, Wang C. Principles of Inter-Amino-Acid Recognition Revealed by Binding Energies between Homogeneous Oligopeptides. ACS Central Sci 2019;5:97-108.

[2] Li X, Guo H, Yang Y, Meng J, Liu J, Wang C, Xu H. A designed peptide targeting CXCR4 displays anti-acute myelocytic leukemia activity in vitro and in vivo. Sci Rep-Uk 2014;4:1-9.

[3] Bai L, Du Y, Peng J, Liu Y, Wang Y, Yang Y, Wang C. Peptide-based isolation of circulating tumor cells by magnetic nanoparticles. Journal of Materials Chemistry B 2014;2:4080-4088.

[4] Zheng Y, Fang X, Yang Y, Wang C. Peptide-directed delivery of drug-loaded nanocarriers targeting CD36 overexpressing cells. Colloids and Surfaces A: Physicochemical and Engineering Aspects 2021;610:125970.

[5] Yu H, Xia D, Zhu Q, Zhu C, Chen D, Gan Y. Supersaturated polymeric micelles for oral cyclosporine A delivery. Eur J Pharm Biopharm 2013;85:1325-1336.

[6] Meng J, Ge Y, Xing H, Wei H, Xu S, Liu J, Yan D, Wen T, Wang M, Fang X, Ma L, Yang Y, Wang C, Wang J, Xu H. Synthetic CXCR4 Antagonistic Peptide Assembling with Nanoscaled Micelles Combat Acute Myeloid Leukemia. Small 2020;16:2001890.
